# Analysis of scientific publications on oral cancer diagnosis delays: a bibliometric study over 22 years

**DOI:** 10.4317/medoral.26719

**Published:** 2024-08-01

**Authors:** Lidiane de Jesus Lisboa, Alessandra Laís Pinho Valente Pires, Adriana Mendonça da Silva, Valéria Souza Freitas

**Affiliations:** 1 ORCID: https://orcid.org/0000-0001-6546-594X. DDS, MSc in Public Health, Department of Health, State University of Feira de Santana, Bahia, Brazil; 2 ORCID: https://orcid.org/0000-0002-6848-8992. DDS, PhD in Public Health, Department of Health, State University of Feira de Santana, Bahia, Brazil; 3 ORCID: https://orcid.org/0000-0001-9314-6970. DDS, PhD in Public Health, Department of Health, State University of Feira de Santana, Bahia, Brazil; 4 ORCID: https://orcid.org/0000-0002-7259-4827. DDS, PhD in Oral Pathology, Department of Health, State University of Feira de Santana, Bahia, Brazil

## Abstract

**Background:**

The area of oncology still lacks bibliometric studies that investigate the diagnostic interval of oral cancer. This study proposed to carry out a bibliometric analysis of epidemiological studies that investigated the diagnosis of oral cancer between 2002 to 2024.

**Material and Methods:**

On April, 2024, the Scopus and Web of Science databases were explored, and the selected articles underwent bibliometric analysis of performance and scientific mapping of journals, authors, institutions, and countries, as well as the predominant topics and trends in research into the diagnosis of oral cancer through an analysis of references and co-occurrence of key words. The analyzes were carried out using the R extension package, Bibliometrix, and the VOSviewer software.

**Results:**

A total of 532 documents were included. China contributed the highest number of publications (36.71%) and total citations [1,584]. Seoane J was the most prolific author [16 (h-index: 9)], while Warnakulasuriya S had the highest total link strength [282 (h-index: 7)] in co-citations. Oral Oncology was identified as the most prolific [231 (72.64%)], co-cited and impactful journal (h-index: 13). Explosions of citations were found for keywords such as “early diagnosis”, “biomarker”, “saliva”, “precancer” and “prognosis”, making it evident that in the field of oral cancer diagnosis there is room for new studies focusing in reducing the diagnostic interval, with the research hotspots being the terms “biomarkers”, “imaging diagnosis” and “gene expression”.

**Conclusions:**

This study provides valuable information that can help researchers and institutions align their research activities according to emerging themes, establish collaborations and allocate resources effectively.

** Key words:**Bibliometric analysis, oral cancer, diagnosis, time intervals, oral squamous cell carcinoma.

## Introduction

Cancer is recognized as a public health problem due to its increasing incidence and mortality, representing a challenge for health systems (1,2). Considered as the third most common cancer in countries with a low or medium human development index (10.2 per 100 thousand) (3), this neoplasm was responsible for 145,353 deaths in 2012, and in 2018, for 177,000 deaths worldwide, representing a 21.8% increase in the number of deaths from the disease in six years (2,4).

Present in around 90% of cases of oral cancer, squamous cell carcinoma is the most common histological type (5,6). Its malignancy potential and survival vary according to the location of the neoplasm and the degree of staging, the latter of which may be influenced by the time interval between the onset of symptoms, their diagnostic confirmation and effective treatment. Therefore, to improve the prognosis of oral lesions, early diagnosis and treatment are essential (7,8).

Frequently used in recent times, bibliometric research, which replaces the classic “statistical bibliographies” (9), uses statistical and mathematical methods to analyze and build indicators on the dynamics and evolution of scientific and technological information on a given topic (10,11). In the area of oncology, bibliometric studies investigated trends in artificial intelligence in this field (12), research related to macrophages in oral cancer (13), bacterial influence on oral cancer (14), poor oral hygiene as a risk factor for oral cancer (15). However, studies that investigate the diagnostic range of oral cancer are still needed. To fill this gap, the purpose of this study was to carry out a bibliometric analysis of epidemiological studies that investigated the diagnosis of oral cancer between the years 2002 and 2024, with a view to answering the following questions:

Q1: How are research articles on oral cancer diagnosis distributed over the years?

Q2: Who are the most prolific and influential nations, institutions, and authors in this field?

Q3: What are the preferred journals for publishing documents related to the diagnosis of oral cancer?

Q4: How is the collaboration network between nations, institutions and authors who have published on the diagnosis of oral cancer?

Q5: What is the research trend and hotspots based on information from published literature?

## Material and Methods

To facilitate replicability, the present study was carried out with the PRISMA structure (16): i) Domain identification with keyword precision; ii) Screening: refinement of the search string and review of results; iii) Eligibility: according to previously defined criteria, and iv) Inclusion: definition of the works that will undergo analysis (Supplement 1). The works were retrieved from Elsevier's Scopus and Clarivate Analytics' Web of Science (WoS) databases, appropriate bases for research in the health area, which meet the quality of eligible literature, as well as the reference format requirement and others. appropriate data for bibliometric analyses.

To search the databases, a Boolean search was applied using the descriptors in two lists with the “AND” operator, for titles, abstracts, and keywords in the case of Scopus and all topics in WoS. The complete search strategy is described in the (Table 1). All searches were carried out independently by two researchers on a single day, April 16, 2024, to avoid significant bias resulting from frequent updating of databases. Any disagreements were resolved through consultation or by enlisting the assistance of external experts to reach a consensus.

Using the “Export” option from WoS and Scopus, the selected results, according to the established criteria, were downloaded with the contents of the record “Full Record and Cited References” in the first and “Citation information, Bibliographical information, Abstract & Keywords, Funding details, Other information” in the second, both in BibTex format. The downloaded files were grouped on the RStudio platform, based on R version 4.3.2, where duplicates were removed and the information from the included articles was saved in two Microsoft Excel 2019 files (Microsoft Corporation, USA), in xle and csv formats. These were later analyzed qualitatively and quantitatively by the R extension package, Bibliometrix (https://www.bibliometrix.org/home/; K-Synth Srl Inc., Naples, Italy), in its open-source interface - Biblioshiny, and by VOSviewer 1.6.19 software (Leiden University, Netherlands). Journal impact factor quartile rankings were derived from the 2022 Journal Citation Reports (JCR).

## Results

The results of the bibliometric analysis, carried out on April 16, 2024, in the SCOPUS database and the Web of Science database, of the terms “oral cancer”, “oral squamous cell carcinoma”, “mouth neoplasms”, “diagnostic delay”, “early diagnosis”, “time intervals”, “diagnostic intervals”, “time factors”, present throughout the document, represented 532 articles, out of a total of 613 returned in the search, respecting the inclusion criteria. 504 articles (82.22%) were retrieved in the SCOPUS database, and 109 articles (17.78%) were in the Web of Science, with 81 duplicate document records being identified and excluded in R Studio. Publications on the diagnosis of oral cancer increased from four articles in 2002 to 62 in 2023, with an average growth rate of 9.59%.

- Contribution of countries

**Figure 1:**
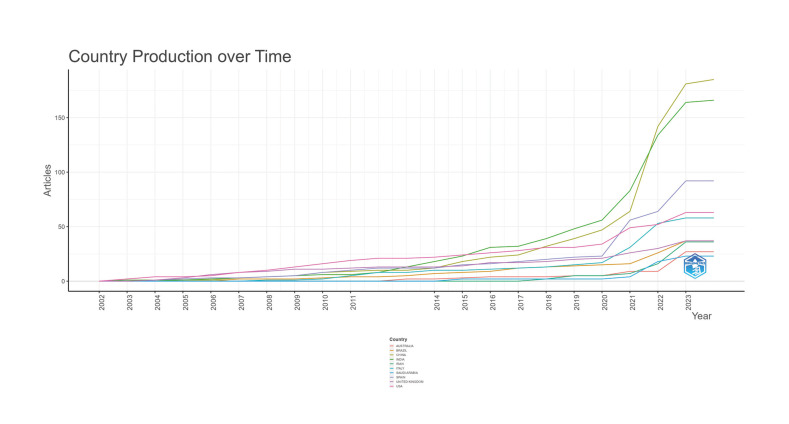
Relative proportion of annual publications. The top 10 country’s change trend in the relative proportion of annual publications on the diagnosis of oral cancer from 2002 to 2024.

All publications covered 54 countries. China published the highest number of studies with 197 (37.03%) documents, followed by India [170 (31.95%)], Spain [92 (17.29%)], United States [63 (11.84%) %)] and Italy [58 (10.90%)]. India and China contributed more than half (68.98%) of the total publications (Supplement 2). The relative proportion of annual publications from 2002 to 2024 from the top 10 countries was presented in Fig. 1. This reveals that at the beginning of the period studied, the United States led in the annual number of publications. However, after 2018 China overtook the United States, and still maintains a growing rate in 2024.

The co-authorship network between countries (This map is available for view online at the link presented in Supplement 3) was analyzed by VOSviewer. A total of 38 countries with a minimum number of three publications were included. As can be seen, the United States and the United Kingdom were situated in a central position of this overlay visualization map. According to the color gradient in the lower right corner, countries such as the United States, Turkey, Netherlands, etc. have been marked with a blue color, which indicates older publications. China, Egypt, United Arab Emirates, etc. were given a yellow color, referring to more recent publications.

- Contribution of authors

**Figure 2:**
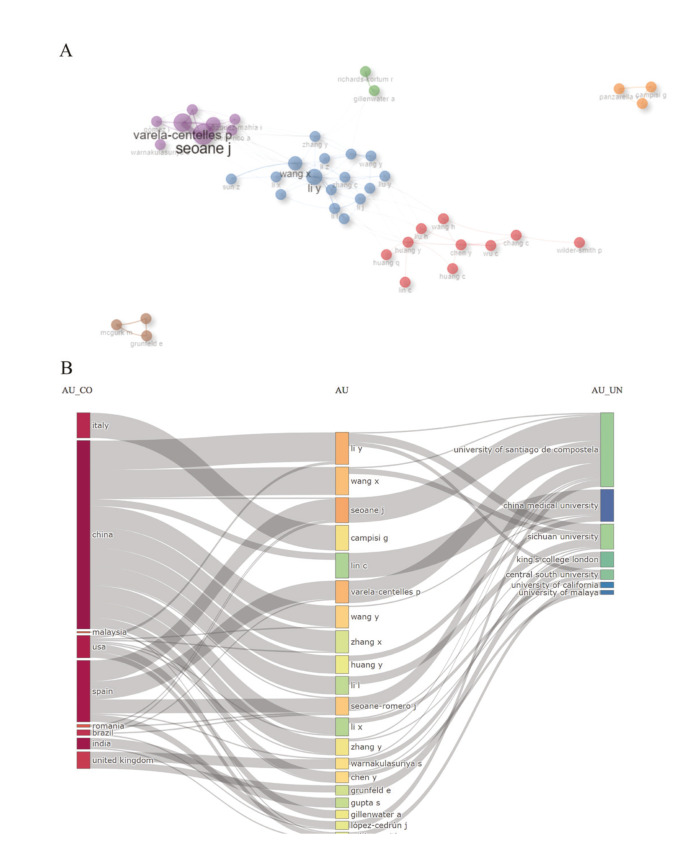
Flow of scientific production and authors’ contributions. (A) Author co-authorship analysis by Biblioshiny. In this network visualization map, each node represents an author, and the lines connecting the nodes represent the co-authorship relationship. The color of the node reflects the cluster that the author participates. (B) Flow of scientific productions between countries, authors, and institutions by Biblioshiny.

2,320 authors were identified over the years under study. Among them, Seoane J was the most prolific author, while Li Y and Warnakulasuriya S are the authors with publications closest to the beginning of the study period. A network for author co-authorship analysis was generated by Biblioshy (Fig. 2). Of the 42 authors included (grouped by the walktrap algorithm), Seoane J. from the University of Santiago de Compostela contributed the largest number of co-authored publications, followed by Varela-Centelles P. from the Galician Health Service, and Li Y from Jilin University. Furthermore, authors with a close cooperative relationship on this map were assigned to a cluster with the same color, and a total of six author clusters were formed. Among them, the cluster of authors from China (blue) has the largest number of authors.

In terms of author co-citation analysis, 21 authors were included with a minimum of 10 citations (This map is available for view online at the link presented in Supplement 3). The top three authors with the highest total link strength (TLS) were Warnakulasuriya S [282], Gonzáles-Moles MA [241] and Seoane J [137]. The flow of scientific productions between countries, authors and institutions can be seen in Fig. 2.

China, Spain and the United States are the countries where the co-authorship of the scientific production of the Correspondences presented in the central column is concentrated. Analyzing the relationship between authors and institutions, the prominence of the University of Santiago of Compostela in this flow becomes clear. In Supplement 4 you can see the impact metrics of the top 20 authors.

- Journals and co-cited journals

**Figure 3:**
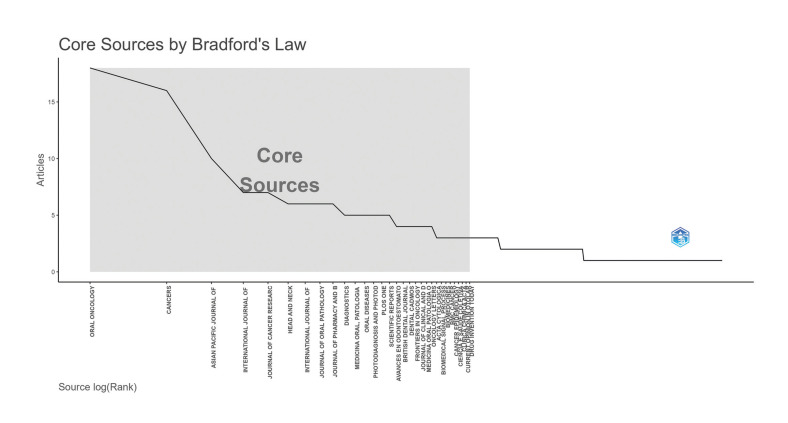
Grouping and co-citation of journals. Grouping of sources using Bradford's Law. The horizontal axis shows the journals, and the vertical axis shows the number of articles published.

The Bradford Law Analysis is presented in Fig. 3, which identifies the main sources of publication within the topic of oral cancer diagnosis. Among the most prolific journals, Oral Oncology [231 documents (72.64%)], Head and Neck [80 (25.15%)] and Asian Pacific Journal of Cancer Prevention [61 (19.18%)] stand out. and 60% were classified as JCR Q1 or Q2. The ranking of the 20 most prolific journals can be seen in Supplement 5.

A co-citation map of journals with a minimum occurrence of 20 citations was generated by VOSviewer (This map is available for view online at the link presented in Supplement 3). There were 53 journals included out of the 318 identified in this study, forming four clusters where Oral Oncology (h-index 13) with a TLS of 8,761 was the most frequently cited journal, followed by Oral Disease (h-index 4), Journal of Oral Pathology & Medicine (h-index 5), Plos One (h-index 4) and Cancer (h-index 6).

- Keywords co-occurrence analysis

**Figure 4:**
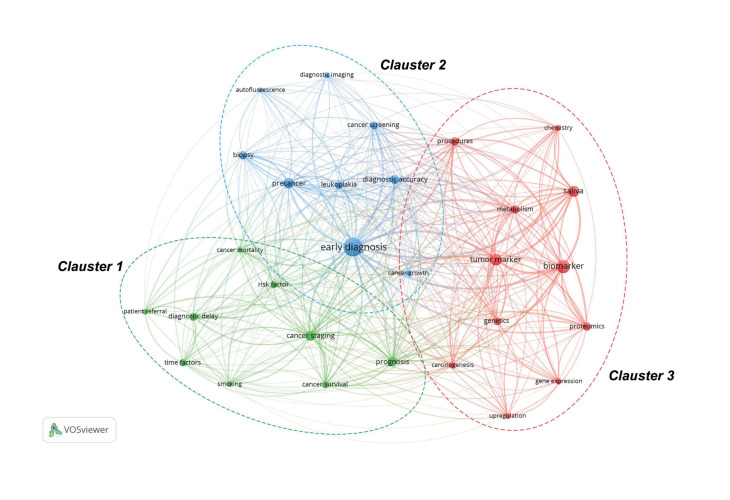
Keyword co-occurrence networks. Network visualization map of keywords co-occurrence analysis. In this network map, each node is a word, the size of the node indicates the number of publications that match the keyword, the links between nodes represent the relationship between the keywords that occurred simultaneously. Keywords with a close relationship are assigned to a cluster with the same color. This map is available for view online at the link <https://tinyurl.com/yl3cxn6w>.

In this study, the keyword co-occurrence network map was constructed with VOSviewer. After manually merging multiple keywords with the same meaning and excluding nonsense keywords, a total of 72 keywords with at least 20 times occurrence were extracted from the 532 publications. Among them, “oral cancer”, “human”, “early diagnosis”, “squamous cell carcinoma” and “oral squamous cell carcinoma” were the five keywords that appeared most frequently. As most of these terms are very general and do not necessarily reflect research interest in the field of oral cancer diagnosis, they were selectively removed from analysis in the VOSviewer software to allow specific topics to emerge. Thus, the topics that emerged were “early diagnosis”, “biomarker”, “saliva”, “precancer” and “prognosis” (Fig. 4).

VOSviewer has a function to automatically divide all keywords into several main groups. As shown in Fig. 4, all keywords can be classified into three clusters as follows: cluster 1 [green nodes, with the term “cancer staging” having the highest link strength [377]], cluster 2 [nodes blue, the term “early diagnosis” showing great link strength (TLS 902)] and in cluster 3 [red nodes, the term “biomarker” leads in occurrences [127] and link strength (TLS 558)]. Observing the trending themes through the keyword co-occurrence overlay visualization map (This map is available for view online at the link presented in Supplement 3) it is observed that “biomarkers”, “imaging diagnosis” and “gene expression” are currently the focus of research attention.

- References and co-cited references

The Spectroscopy analysis of the Reference Publication Year (RPY) showed that 2020, 2021 and 2019 are the years that, respectively, have the best representation in scientific production, in the period under study. The 20 most cited publications in the field of oral cancer diagnosis were presented in Table 2. Of these, the majority of articles were reviews, and all were cited more than 93 times, with the first of them, a case-control study conducted by Li Y and collaborators in 2004, having a rate of 22.15 citations per year.

## Discussion

Mapping science is of great value to scholars from all areas, as it presents an overview of the voluminous scientific production that is dispersed across several bibliographic databases. Thus, it allows analyzing the structure of the literature and uncovering the evolutionary nuances, trends, and relevant topics, while also shedding light on the emerging areas of this field (11,17), as well as assisting in decision making (18). Numerous studies are conducted addressing the diagnosis of oral cancer, which suggests a high level of global interest, which reflects the positive average growth rate of publications in this area in the period studied. Corroborating other bibliometric research that investigated different topics related to cancer, where they found positive rates for the growth of publications (17,19,20).

In the present study, the analysis of production by countries reveals that progress in this field of research has been driven by countries in Asia, Europe, and North America, mainly China, Spain, and the USA, respectively. China leads in total citations, followed by the United States and the United Kingdom, corroborating other authors (12,13,19). However, the average number of citations per document in China places it in 8th place in this study, behind countries such as Spain, Italy, and Australia. Different factors such as the number of institutions and researchers, the availability of resources and patients, can contribute to these findings. Likewise, the high incidence of oral cancer in some of these countries (3,4) and its impact on survival highlights the challenge of diagnosis in the shortest interval (8,21), making research in this area a priority by organizations such as the World Health Organization and other institutions.

Observing the network map co-authored by the authors, we can see through the centrality of the node how much it is interconnected to other nodes. Seoane J. from the University of Santiago de Compostela in Spain is first in co-authorship and third in co-citation, a fact that justifies the prominence of the University to which the author is affiliated in the flow of scientific productions. Li Y. from Jilin University in China is the second most prolific author in terms of number of publications and is among the three with the most co-authorships. These results suggest that the main authors contributed high-impact articles in this area, and that their studies may continue to influence the development of the field of oral cancer diagnosis, both from a quantitative and qualitative point of view in the future.

Bradford's Law refers to the dispersion of authors in different scientific journals, allowing us to determine the journal that most concentrates articles on a given area (22). According to this law, in this study, the journal Oral Oncology led the ranking with the largest number of publications on the topic. This same newspaper was also the most cited and most impactful, similar to other studies (13,15). Because Oral Oncology is an oncological journal, most of the articles published are naturally related to cancer research. Furthermore, the type and quality of published documents, the number of articles per edition, number of editions per year, may have contributed to these findings (23,24). The majority of journals presented in the results of this search are classified as Q1 or Q2, which indicates that well-designed, high-quality studies form the basis for the field of oral cancer diagnosis.

The co-occurrence analysis of article keywords over the years can reflect the current research focus, help understand the evolution trend, and predict the research perspective and hotspot in the future (25). Thus, it is noted that in this study the direction of current research in the field of oral cancer diagnosis focuses on articles on “biomarkers”, “imaging diagnosis” and “gene expression”, revealing that researchers are searching of methods to reduce the time interval until diagnosis and initiation of treatment, since studies point to their negative impact on the individual's survival (8,21), on costs for treatment (20,26) and quality of life of oral cancer survivors (27,28).

In the same sense, the co-citation analysis of references in this study reinforces the themes found in the co-occurrence of key words and indicates the most relevant works in the area of oral cancer diagnosis, which generally provide a knowledge base from which subsequent articles are guided (3,29-32).

This study has limitations that mainly involve the limits of the search: i) although two databases were used, not all the literature related to the researched field was obtained due to the diversity of keywords; ii) several recently published, and potentially high-impact research studies may not be included in the analysis due to low citation frequency. These limitations could be mitigated with the use of logical operators in the search strategy to increase the precision of the retrieved results. However, different results can be found with updates to bibliometric data or searches in other databases.

The results of this study indicate that the field of oral cancer diagnosis presents prospects for future expansion. It is currently encouraged by China, the leading country in terms of publications and citations in the area. Researchers interested in the topic must strengthen cooperation between different countries in order to build more relevant evidence. The journal Oral Oncology was the main source of publication of articles in terms of publication, citation and impact in the period considered. Within the area under study there is room for new research focused on reducing the diagnostic interval, identifying hotspots such as “biomarkers”, “imaging diagnosis” and “gene expression”.

This bibliometric study provides a comprehensive analysis of the literature in the field of oral cancer diagnosis, which can guide researchers in planning future projects, better inform healthcare professionals about research trends, and provide useful references for scholars and policymakers in this area. Furthermore, these results can help institutions align their research activities according to emerging themes, establish collaborations, prioritize, and allocate resources effectively.

## Figures and Tables

**Table 1:** Complete search strategy for Scopus and Web of Science (WoS).

DATA SCOURCES	SEARCH STRATEGIES
Scopus	(TITLE-ABS-KEY (("Oral cancer" OR "oral squamous cell carcinoma" OR "mouth neoplasms")) AND TITLE-ABS-KEY (("diagnostic delay" OR "Early diagnosis" OR "time intervals" OR "diagnostic intervals" OR "time factors")))
WoS	TS= (("Oral cancer" OR "oral squamous cell carcinoma" OR "mouth neoplasms")) AND TS= (("diagnostic delay" OR "Early diagnosis" OR "time intervals" OR "diagnostic intervals" OR "time factors")

**Table 2:** The 20 most cited publications in the field of oral cancer diagnosis in the last 22 years.

PAPER	DOI	TC ^*^	TC PER YEAR	NORMALIZED TC
LI Y, 2004, Clin Cancer Res	10.1158/1078-0432.CCR-04-1167	443	21.10	2.91
TIZIANI S, 2009, Neoplasia	10.1593/neo.81396	213	13.31	3.92
JEYARAJ PR, 2019, J Cancer Res Clin Oncol	10.1007/s00432-018-02834-7	183	30.50	8.69
AUBREVILLE M, 2017, Sci Rep	10.1038/s41598-017-12320-8	179	22.38	5.79
MAITI KK, 2012, Nano Today	10.1016/j.nantod.2012.02.008	163	12.54	3.92
KAH JCY, 2007, Int J Nanomed	NA^ †^	160	8.89	3.60
KHURSHID Z, 2018, Adv Clin Chem	10.1016/bs.acc.2018.05.002	150	21.43	6.79
LU YC, 2012, Cancer Prev Res	10.1158/1940-6207.CAPR-11-0358	147	11.31	3.53
MEHROTRA R, 2011, Head Neck Oncol	10.1186/1758-3284-3-33	141	10.07	4.66
GÓMEZ I, 2010, Oral Dis	10.1111/j.1601-0825.2009.01642.x	133	8.87	2.62
FEDELE S, 2009, Head Neck Oncol	10.1186/1758-3284-1-5	133	8.31	2.45
GÓMEZ I, 2009, Eur J Oral Sci	10.1111/j.1600-0722.2009.00672.x	132	8.25	2.43
GÜNERI P, 2014, Oral Oncol	10.1016/j.oraloncology.2014.09.005	123	11.18	3.37
SCULLY C, 2008, Am J Dent	NA ^?^	116	6.82	2.39
ONIZAWA K, 2003, Oral Oncol	10.1016/S1368-8375(03)00075-7	116	5.27	2.01
WANG Q, 2014, Sci Rep	10.1038/srep06802	113	10.27	3.10
MARAKI D, 2004, J Oral Pathol Med	10.1111/j.1600-0714.2004.0235.x	100	4.76	0.66
WELIKALA RA, 2020, Ieee Access	10.1109/ACCESS.2020.3010180	98	19.60	4.13
SCOTT SE, 2005, Oral Oncol	10.1016/j.oraloncology.2004.10.010	97	4.85	2.14
ABATI S, 2020, Int J Environ Res Public Health	10.3390/ijerph17249160	93	18.60	3.92

Sort in descending order, according to the total number of citations. ^* ^Total citations. † No identification.
